# Illumina-based analysis yields new insights into the diversity and composition of endophytic fungi in cultivated *Huperzia serrata*

**DOI:** 10.1371/journal.pone.0242258

**Published:** 2020-11-19

**Authors:** Shipeng Fan, Liyun Miao, Haodong Li, Aihua Lin, Fajun Song, Peng Zhang

**Affiliations:** 1 Hubei Provincial Key Laboratory for Protection and Application of Special Plants in Wuling Area of China, Key Laboratory of State Ethnic Affairs Commission for Biological Technology, Center for the Conservation and Utilization of Medicinal Plant Resources, College of Life Science, South-Central University for Nationalities, Wuhan, China; 2 College of Basic Medical Sciences, Shanxi University of Traditional Chinese Medicine, Jinzhong, China; Friedrich Schiller University, GERMANY

## Abstract

Endophytic fungi play an important role in plant growth. The composition and structure of endophytes vary in different plant tissues, which are specific habitats for endophyte colonization. To analyze the diversity and structural composition of endophytic fungi from toothed clubmoss (*Huperzia serrata*) that was artificially cultivated for 3 years, we investigated endophytic fungi from the roots, stems and leaves using comparative sequence analysis of the ITS2 region of the fungal rRNA genes sequenced with high-throughput sequencing technology. Seven fungal phyla were identified, and fungal diversity and structure varied across different tissues, with the most distinctive community features found in the roots. A total of 555 operational taxonomic units (OTUs) were detected, and 198 were common to all samples, and 43, 16, 16 OTUs were unique to the root, stem, leaf samples, respectively. Taxonomic classification showed that Ascomycota and Basidiomycota were dominant phyla, and *Cladosporium*, *Oidiodendron*, *Phyllosticta*, *Sebacina* and *Ilyonectria* were dominant genera. The relative abundance heat map at the genus level suggested that *H*. *serrata* had characteristic endophytic fungal microbiomes. Line discriminant analysis effect size analysis and principal coordinate analysis demonstrated that fungal communities were tissue-type and tissue-site specific. Overall, our study provides new insights into the complex composition of endophytic fungi in *H*. *serrata*.

## Introduction

*Huperzia serrata*, a traditional medicinal plant in China, has bioactive properties favorable for treating fever, schizophrenia, and myasthenia gravis [1, 2]. Huperzine A isolated from *H*. *serrata* has a potent anti-acetylcholinesterase (AChE) activity [3], which has been approved in China as a drug to treat Alzheimer’s disease [4] and is currently used as a supplement for preventing further memory degeneration in USA [5]. However, wild *H*. *serrata* shows a low huperzine A content (ca. 0.007%), a limited geographic distribution, and an extremely slow growth [6]. Meanwhile, the excessive mining of wild *H*. *serrata* has degraded their habitat [2]. Wild *H*. *serrata* populations are insufficient to meet market demand. Therefore, it is very important and necessary to develop artificial cultivation of *H*. *serrata*, however there are still many challenges to growing *H*. *serrata* in a non-wild setting.

Along with emerging research on plant-microbe interactions, accumulating evidences suggest that endophytic fungi play important roles in plant growth [7, 8]. Some endophytic fungi can benefit plants by producing plant hormones [7], improving stress resistance [8], protecting plants from phytopathogens [9], and enabling nutrient uptake [10]. Previous studies examining the endophytic fungi of *H*. *serrata* and their secondary metabolic products revealed that they exhibited various biological activities, including antimicrobial activity, acetylcholinesterase inhibitory activity, nematocidal activity, and inhibition of nitric oxide production, among others [11–14]. In addition, some of endophytic fungi in *H*. *serrata* can produce huperzine A [15–17]. The distribution of some endophytic fungi in the host plants exhibited tissue specificity, which is an important influencing factor for accumulation of bioactive substances in different tissues [18, 19]. Although there are many studies focusing on endophytes of *H*. *serrata*, the understanding of the endophytic community in different tissues associated with *H*. *serrata* is still limited. Therefore, it is necessary to study the diversity and composition of endophytes in different tissues within *H*. *serrata*.

The Illumina-based high-throughput sequencing technology can comprehensively reveal the diversity and composition of plant-associated endophytes [20]. Lee *et al*. used high-throughput sequencing technology to reveal diversity of endophytic bacterial, archaeal and fungal communities inhabiting different rhizocompartments of tomato plants in real-world environments [21]. Chen *et al*. used high-throughput sequencing technology to analyze and compare the endophytic fungal community structures associated with stems and roots of *Dendrobium huoshanense* [22]. However, few researches on the diversity and composition of endophytes in *H*. *serrata* based on high-throughput sequencing have been conducted.

In the present study, the Illumina-based high-throughput sequencing analysis of the ITS2 region of fungal ribosomal RNA (rRNA) genes was conducted to describe the diversity and composition of endophytic fungi in the various tissues of *H*. *serrata*. As far as we know, this is the first time that the high-throughput amplicon sequencing has been used to study fungal community structure and diversity in *H*. *serrata*. Our results provide new insights into the fungal communities and lay a foundation for further study of *H*. *serrata*.

## Materials and methods

### Plant materials and treatments

Healthy three-year-old artificially cultivated *H*. *serrata* plants were randomly collected in July 2019 from the medicinal plant plantation in Huayuan County, Xiangxi Tujia and Miao Autonomous Prefecture, Hunan Province, China. These collected plants were developed from the mature spores of *H*. *Serrata* in the plantation, which guaranteed the exact growth age of them, and were properly managed, including watering, weeding and deworming. All plant samples were placed in aseptic bags that were placed on ice and immediately transported back to our laboratory, and three plants were randomly selected for investigation. After removing all sporangia, the root (marked as R1, R2, R3), stem (marked as S1, S2, S3) and leaf (marked as L1, L2, L3) samples of all three plants were separately collected using a sterile scissors and surface-sterilized using a series of washing steps: 70% (v/v) ethanol for 1 min, 3% (v/v) sodium hypochlorite solution for 3 min, 2.5% (w/v) sodium thiosulfate for 5 min, and rinsing the samples five times with sterile water [21]. Subsequently, nine samples from the root, stem and leaf tissues of three *H*. *Serrata* plants were used separately to extract the fungal genome DNA within them.

### DNA extraction, PCR amplification, and ITS clone library construction

The root, stem, and leaf samples were rapidly ground to fine powder with liquid nitrogen in a sterilized and pre-cooled mortar. The resulting powder was then transferred to a bead tube for total DNA extraction using a MN NucleoSpin 96 Soil DNA kit (Macherey-Nagel, Dueren, Germany) according to the manufacturer instructions. DNA was stored at −20°C until subsequent analysis. The target-specific primers ITS1F (5'-CCTGGTCATTTAGAGGAAGTAA-3'), ITS4 (5'-TCCTCCGCTTATTGATATGC-3'), and fITS7 (5'-GTGARTCATCGAATCTTTG -3'), which do not amplify the chloroplast or mitochondrial rRNA genes of *H*. *serrata*, were used to amplify the ITS region of the fungal rRNA genes. PCRs (50 μL) were assembled and conducted using a reaction program as follows: 5 min at 94°C, 35 cycles of 1 min at 94°C, 50 sec at 54°C, and 60 sec at 68°C, then 10 min at 68°C. The PCR products were examined on a 1.8% agarose gel and purified using an AxyPrep DNA Gel Extraction Kit (Axygen Biosciences, Union City, CA, United States). Then, the construction and sequencing of the ITS clone libraries were performed by Beijing Biomarker Biotechnology Co., Ltd (Biomarker Biotechnology, Beijing, China) using an Illumina HiSeq 2500 platform.

### Sequence processing and data analysis

FLASH software (v1.2.11) [23] was used to splice the reads of each sample through overlap, and the resulting spliced sequences were used as raw tags. Trimmomatic software (v0.33) [24] was used to filter the spliced raw tags to obtain high-quality tags (clean tags). UCHIME software (v8.1) [25] was used to identify and remove chimeric sequences to obtain the final effective tags, which were further clustered into operational taxonomic units (OTUs) with 97% pairwise identity.

The representative sequences of OTUs were used to perform taxonomic analysis through aligned to the UNITE database using QIIME software (v1.7.0) [26]. QIIME software was also used to select the OTU sequence with the highest abundance at the taxonomic level of the genus, carry out multiple sequence alignment, construct the phylogenetic tree, and then create a graph with the Python language tool. R software was used to obtain Venn diagrams and microbial community bar plots to characterize the richness of a specific fungal community.

Alpha diversity indexes, including Chao1, Ace, Shannon and Simpson, were evaluated using Mothur software (version v.1.30) [27]. In addition, OTU coverage was also counted to determine whether the sequencing results were representative of the actual microbial communities in our samples. Rarefaction curves, reflecting the sequencing depth, were calculated and constructed using QIIME software.

Beta diversity indexes were evaluated using QIIME software to compare the similarity of different samples in species diversity. Principal Coordinate Analyses (PCoA) utilizing the Bray-Curtis distances were used to observe the relationships between fungal community structures in different tissues. Analysis of Unweighted Pair-Group Method with Arithmetic Means (UPGMA) was carried out to determine whether the samples had significant microbial community differences in a UPGMA tree. The heat map of genera differences between groups was drawn based on the OTU-Table to understand the fungal community composition at genus level among different tissues. Furthermore, Line discriminant analysis effect size (LEfSe) analysis [28] using Galaxy online (http://huttenhower.sph.harvard.edu/galaxy/) was used to identify differentially abundant features among samples for biomarker discovery.

## Results

### Characteristics of sample sequences

**Fig 1:**
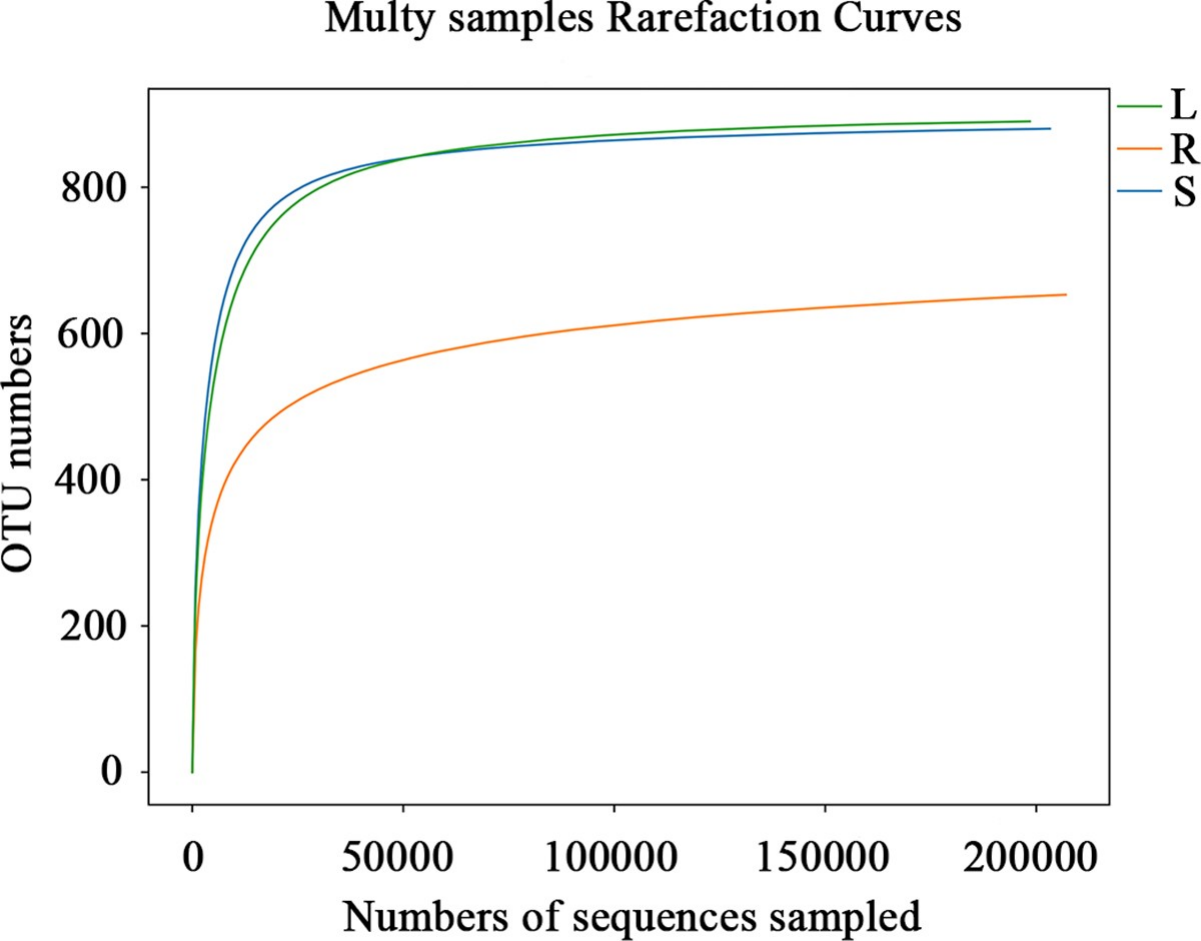
Rarefaction curves based on the ITS2 sequences of endophytic fungi from the root (R), stem (S) and leaf (L) samples associated with *H*. *serrata*.

The quality of the sequencing data was evaluated mainly through the statistics of sequence number, sequence length, GC content, Q20 and Q30 quality values, effective ratio, and other parameters in each stage (S1 Table). The number of effective tags per sample ranged from 230,078 to 231,811, and the average length of sequences from the root, stem and leaf samples was 304, 303 and 299 bp, respectively, however the length of sequencing tags in the three tissue samples mainly fell within the range of 280–370 bp (S1 Fig). The rarefaction curves (Fig 1) tended to be flat, indicating that our sequencing depth was sufficient. Similarly, more than 0.999 coverage suggested that the ITS libraries were large enough to capture most of the fungal diversity in the samples used in this study (Table 1).

**Table 1:** The richness and diversity indexes of endophytic fungi from the root, stem and leaf samples associated with *H*. *serrata*.

Sample origin	OTUs	Total OTUs	ACE	Chao1	Simpson	Shannon	Coverage
Roots	284	555	314.5561	350.1111	0.051	3.4706	0.9998
Stems	465		467.8932	466.5	0.0321	4.1734	1
Leaves	484		501.5221	507.25	0.0475	3.8822	0.9999

### Richness and diversity of endophytic fungi

**Fig 2:**
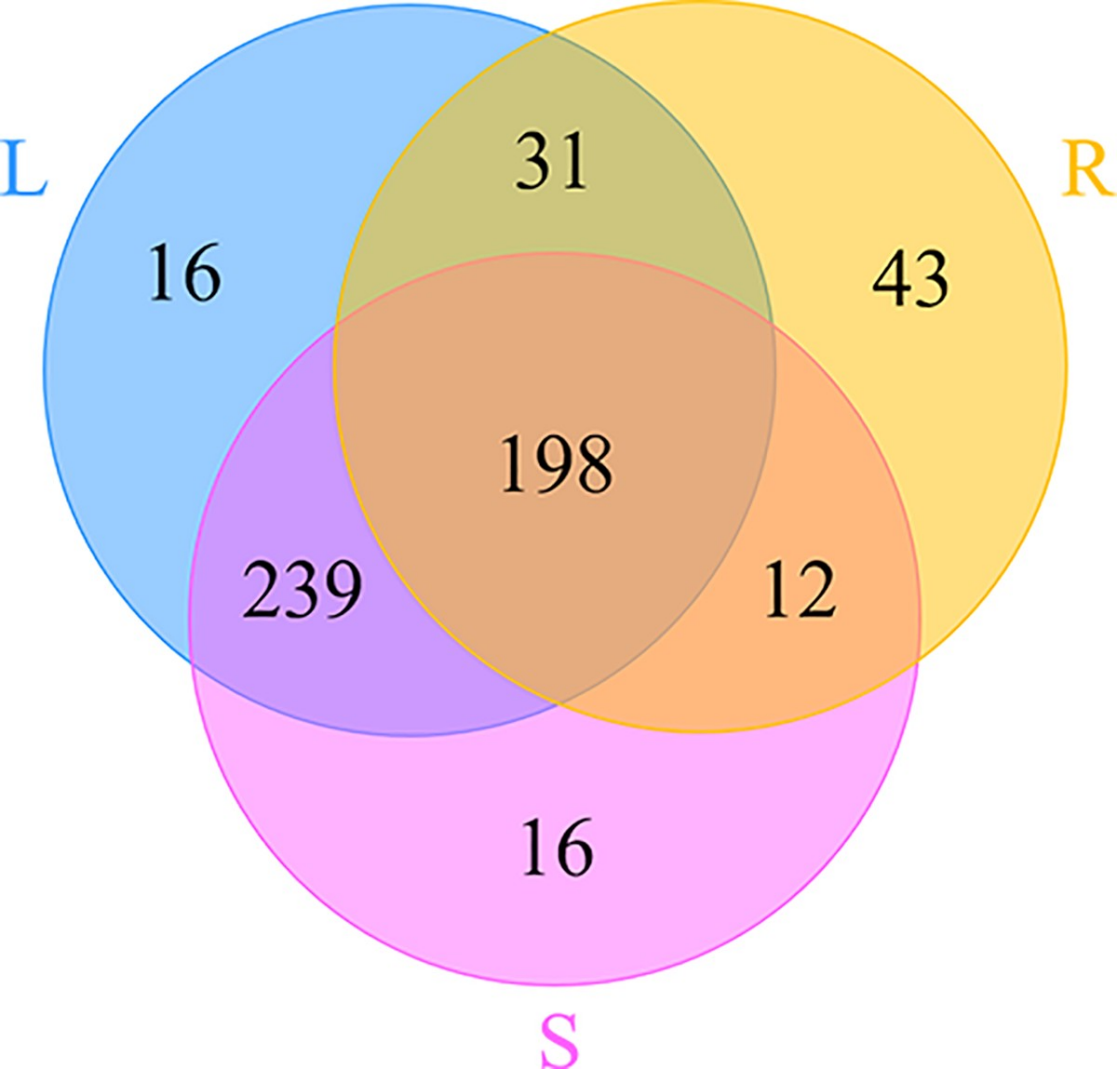
Venn diagram showing the OTUs shared among the root (R), stem (S) and leaf (L) samples associated with *H*. *serrata*.

A total of 555 OTUs were detected across all of the ITS libraries, including 284 OTUs in the root samples, 465 OTUs in the stem samples and 484 OTUs in the leaf samples (Table 1). Among them, 198 OTUs were common to all samples, and 43, 16 and 16 OTUs were exclusive to the root, stem and leaf samples, respectively (Fig 2). The Chao1 and Ace indexes showed that the community richness of the endophytic fungi in the leaves was higher than that in the stems and roots (Table 1). However, the Shannon and Simpson indexes showed that the community diversity of the endophytic fungi in the stems was highest, followed by the leaves and roots (Table 1).

### Taxonomic distribution of endophytic fungi

**Fig 3:**
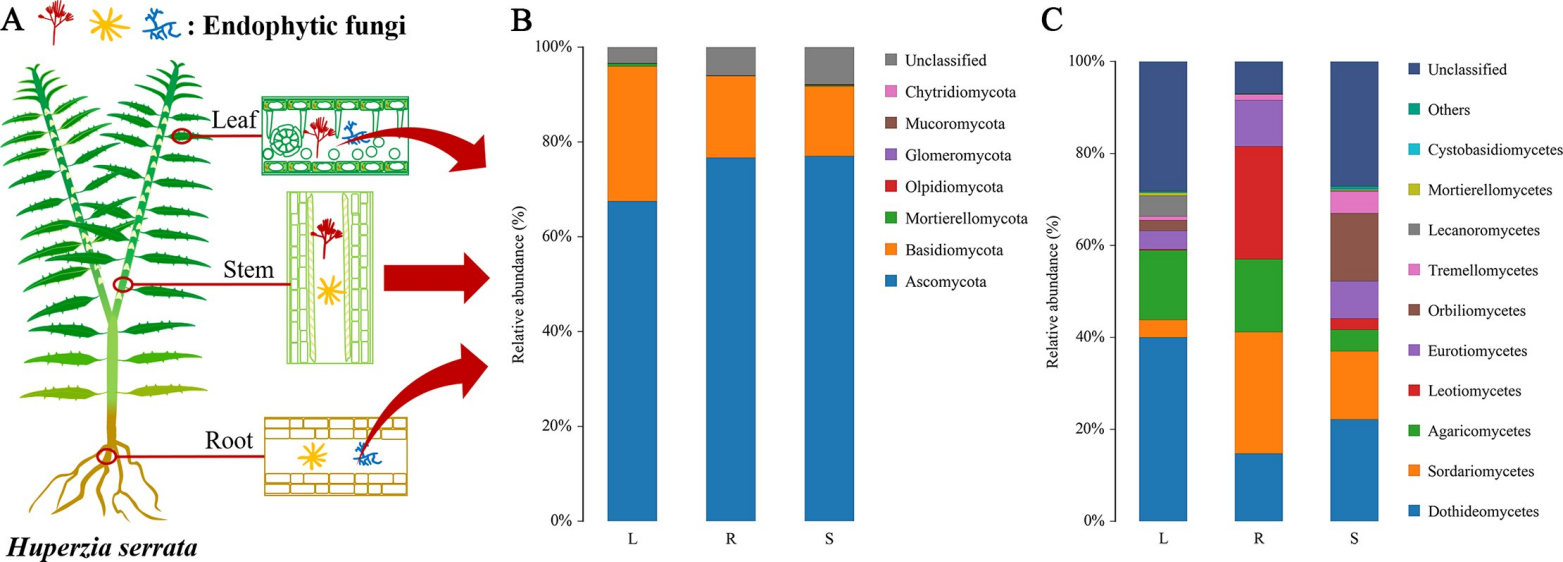
Distribution of endophytic fungi in *H*. *serrata* (A) and relative abundances of endophytic fungi at the phylum (B) level and class (C) level in the root (R), stem (S) and leaf (L) samples of *H*. *serrata*.

The taxonomic distribution of endophytic fungi in the roots, stems and leaves of *H*. *serrata* was displayed in Fig 3. After screening out rare OTUs, the remaining OTUs represented 7 phyla, 21 classes, 50 orders, 95 families, and 120 genera (Table 2). The 7 identified phyla were Ascomycota, Basidiomycota, Chytridiomycota, Glomeromycota, Mortierellomycota, Mucoromycota, and Olpidiomycota, and the relative abundances of these phyla varied across the three tissues (Fig 3B). Among them, Ascomycota and Basidiomycota were predominant phyla, accounting for 71.9% and 13.2% of sequences, respectively, while the other phyla were all below 1% of sequences (Table 3). In addition, all the phyla were found in the leaf samples, but Chytridiomycota and Glomeromycota were absent in the root samples and the stem samples, respectively.

**Table 2:** Numbers of taxa of endophytic fungi in the root, stem and leaf samples of *H*. *serrata*.

**Sample origin**	**Phylum**	**Class**	**Order**	**Family**	**Genus**
Roots	6	14	30	61	80
Stems	6	20	48	91	113
Leaves	7	20	49	90	110
Total	7	21	50	95	120

**Table 3:** Distribution of fungal microbiome at phylum level in the root, stem and leaf samples of *H*. *serrata*.

Taxon	Relative abundance (%)		
	Roots	Stems	Leaves
Ascomycota	76.67	76.31	65.07
Basidiomycota	17.29	15.05	28.48
Chytridiomycota	0	0.01	0.004
Glomeromycota	0.07	0	0.0005
Mortierellomycota	0.01	0.18	0.60
Mucoromycota	0.0004	0.03	0.02
Olpidiomycota	0.001	0.16	0.02
Unclassified	5.95	8.26	5.80

In detail, 21 classes belonging to the 7 phyla were identified (S2 Table). Among them, 20 classes were identified in the leaf and stem samples, respectively, while only 14 classes were identified in the root samples. The predominant classes (top 10) were Dothideomycetes, Sordariomycetes, Eurotiomycetes, Tremellomycetes, Leotiomycetes, Agaricomycetes, Mortierellomycetes, Cystobasidiomycetes, Ustilaginomycetes, and Orbiliomycetes in all samples (Fig 3C). However, class distributions differed greatly across the three tissues (S2 Table). For example, GS18 was not found in the leaf samples, Glomeromycetes was not found in the stem samples, and Pezizomycetes, Agaricostilbomycetes, Cystobasidiomycetes, Exobasidiomycetes, Utilaginomycetes, Spizellomycetes, GS17 were not found in the root samples (S2 Table).

The top 26 genera (i.e., those with relative abundance > 0.2%) were selected to make a heatmap clustering (S2 Fig and Table 4), which further indicated that species distributions differed greatly across the three tissue samples. Among them, 20 genera belonged to Ascomycota, while 5 belonged to Basidiomycota and 1 belonged to Mortierellomycota. The genera of *Oidiodendron*, *Ilyonectria*, *Chloridium*, *Russula*, *Sebacina*, *Cladophialophora*, *Periconia*, *Pezicula*, *Roussoella*, *Scytalidium*, *Dactylonectria*, *Papiliotrema*, *Pochonia*, and *Verticillium* were mainly distributed in the roots. The genera of *Cladosporium*, *Saitozyma*, *Pyrenochaetopsis*, *Claviceps*, *Cyphellophora*, and *Purpureocillium* were mainly distributed in the stems, while the dominant genera in the leaves included *Phyllosticta*, *Serendipita*, *Devriesia*, and *Mortierella*. In addition, *Botryosphaeria*, *Scytalidium*, and *Idriella* were the exclusive genera in the root samples, while *Phialophora*, *Lecophagus*, *Clavaria*, and *Peniophora* were exclusive to the stem samples, and no genus was exclusive to the leaf samples (S3 Table).

**Table 4:** Distribution of fungal microbiome at genus level (relative abundance > 0.2%) in the root, stem and leaf samples of *H*. *serrata*.

Phyla	Class	Genus	Relative abundance (%)		
			Roots	Stems	Leaves
Ascomycota	Dothideomycetes	*Phyllosticta*	0.02	0.07	11.64
		*Cladosporium*	2.55	6.52	5.74
		*Devriesia*	0.001	0.20	0.66
		*Pyrenochaetopsis*	0.10	2.29	1.51
		*Periconia*	2.77	0.02	0.02
		*Roussoella*	1.85	1.23	0.08
	Eurotiomycetes	*Cyphellophora*	0.001	1.03	0.89
		*Cladophialophora*	6.54	0.50	2.14
	Leotiomycetes	*Pezicula*	2.70	0.001	0
		*Scytalidium*	1.73	0	0.001
		*Oidiodendron*	11.55	1.97	0.06
	Sordariomycetes	*Chloridium*	8.38	0.10	0.01
		*Verticillium*	0.47	0.09	0.29
		*Clonostachys*	0.28	0.27	0.20
		*Claviceps*	0.0004	1.56	0.12
		*Pochonia*	0.48	0.13	0.02
		*Trichoderma*	0.50	0.72	0.02
		*Dactylonectria*	1.59	0.004	0.001
		*Ilyonectria*	9.70	0.02	0.04
		*Purpureocillium*	0.02	0.41	0.16
Basidiomycota	Agaricomycetes	*Russula*	8.29	0	0.001
		*Sebacina*	7.27	4.12	0.31
		*Serendipita*	0.0004	0.003	1.38
	Tremellomycetes	*Papiliotrema*	0.50	0.09	0.07
		*Saitozyma*	0.73	3.30	0.12
Mortierellomycota	Mortierellomycetes	*Mortierella*	0.01	0.18	0.60

In addition, the endophytic fungi of the root samples possessed a better taxonomic annotation of their OTUs and harbored a minimum proportion of unclassified OTUs. For examples, the percentage of classified taxa at the class level in the roots were 85.46%, which were higher than that in the stems (72.13%) and leaves (59.04%), while 61.65% classified taxa at genus level in the roots were higher than 29.97% in the stems and 28.84% in the leaves (Fig 3C and S3 Fig).

### Comparative analysis of endophytic fungi

**Fig 4:**
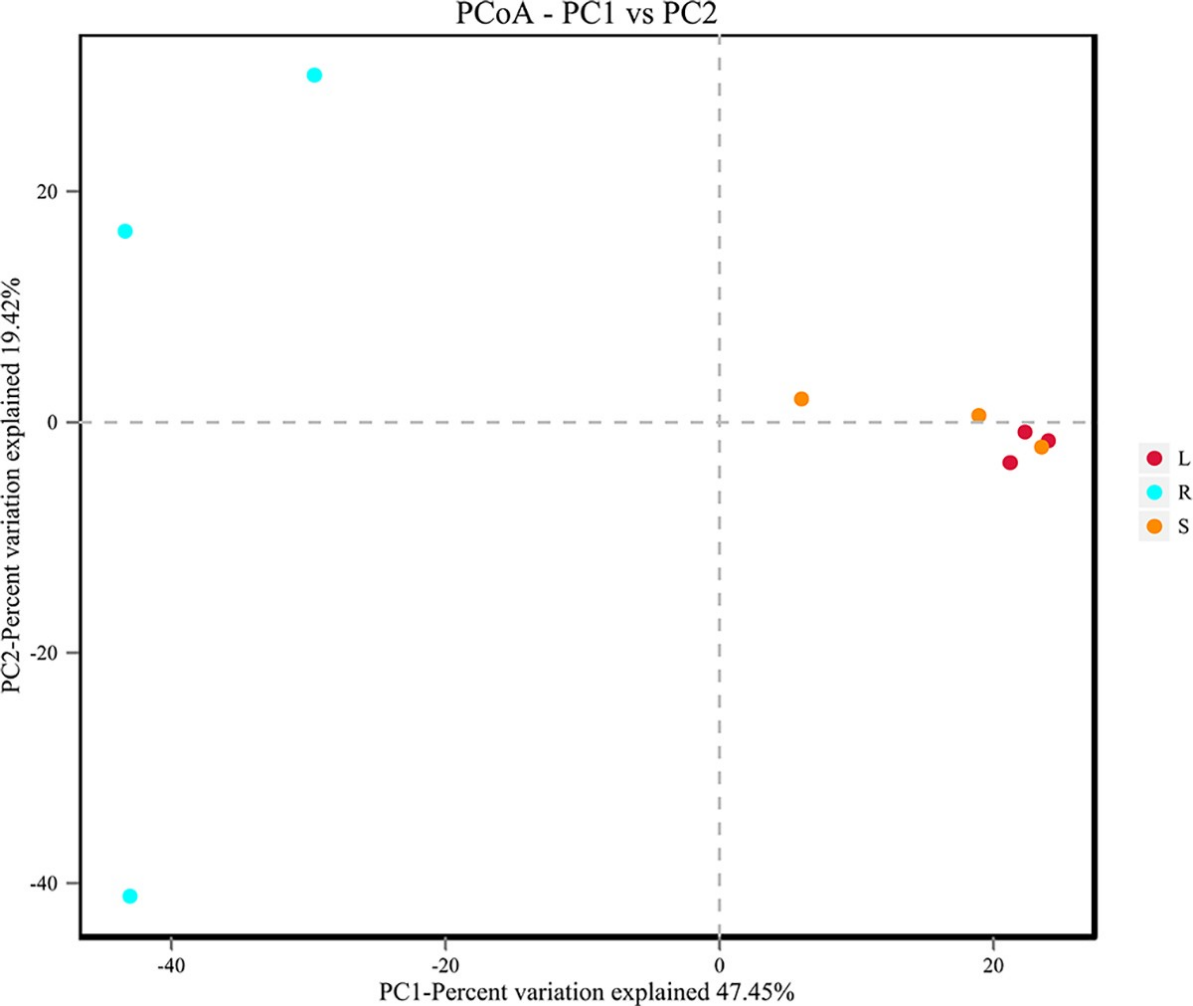
Comparison of microbial communities in the root (R), stem (S) and leaf (L) samples of *H*. *serrata* based on Bray-Curtis.

Important distinctions were found in the composition of fungal communities in the root, stem and leaf samples. Two different clusters were observed at the genus level in the UPGMA tree: the fungal microbiota from *H*. *serrata* leaves and stems clustered together, but the roots formed their own cluster and distinctly separated from those of the stems and leaves (S4 Fig), suggesting that the endophyte microbiomes of the leaf samples were more similar to that of the stem samples than to that of the root samples, and that the endophytic fungi of the leaf and stem samples might share a same origin. PCoA revealed the main variations in fungal community composition among the three tissues (Fig 4), and the highest variations in the microbiota of different samples were 25.99% (PC1) and 17.25% (PC2), representing a strong separation based on the plant tissues.

**Fig 5:**
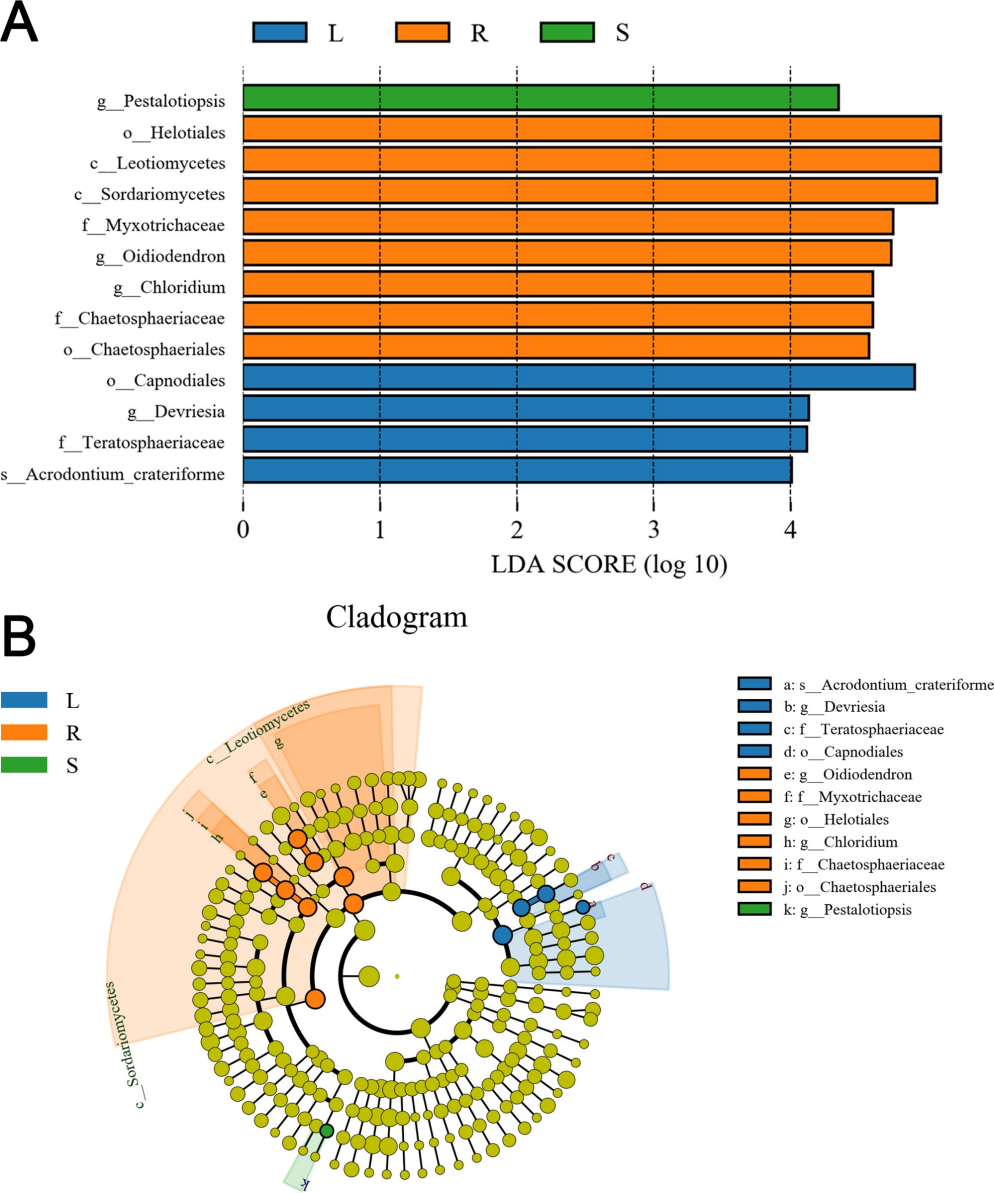
Groups from the phylum-to-species level determined to be significant representatives of their sample type based on LEfSe analysis. (A) The figure shows the taxa with an LDA score greater than 4.0. The length of the histogram represents the influence of different taxa (LDA score), and different colors represent different grouped taxa. (B) The cladogram represents the taxonomic hierarchical structure of the identified habitat biomarkers generated using LEfSe. The circles radiating from the inside to the outside of the branching diagram represent the taxonomic levels from the phylum to the species; each small circle at different classification levels represents a classification at this level, and the diameter of the small circle is proportional to the relative abundance. Taxa with no significant differences are shown in yellow, while taxa with significant differences are colored according to the grouping of the most abundant taxa.

Significantly different taxon abundances of endophytic fungi were found among the three tissues, as determined by LEfSe (Fig 5). At the genus level, *Pestalotiopsis* was significantly enriched in the stems, while *Oidiodendron* and *Chloridium* were more abundant in the roots. *Hannaella* exhibited relatively higher abundance in the leaves than in the roots and stems. These differentially abundant taxa can be considered as potential biomarkers (line discriminant analysis (LDA) score > 4, *P* < 0.05).

## Discussion

In this study, the endophytic fungi associated with the root, stem and leaf tissues of three-year-old *H*. *serrata* were characterized and compared. Alpha diversity analysis (Chao1, Ace, Shannon, Simpson) indicated that richness and diversity of endophytic fungi in the *H*. *serrata* root samples were lowest (Table 1), which were different from those of the *D*. *huoshanense* roots [22], that have a higher richness and diversity of endophytic fungi. A total of 555 fungal OTUs were detected from the three tissue samples of *H*. *serrata*, and were further classified into 7 phyla, 21 classes, 50 orders, 95 families and 120 genera. However, there were still some OTUs sequences were not taxonomically annotated at different taxon level, such as 38.35% unclassified taxa (OTUs) at genus level in the root samples (Fig 3C, S3 Fig), indicating that we still lacked the full knowledges of endophytic fungi of the investigated *H*. *serrata* in nature.

The distribution of endophytic fungal taxa varied greatly among different plant tissues, which might be related to host genotype [29], growing environment [30], and plant age [31]. The root, stem, and leaf tissues of the investigated *H*. *serrata* were colonized by some of the same fungal communities, but in different proportions. Ascomycota and Basidiomycota were the most abundant phyla in all of the three tissues of *H*. *serrata*, which were consistent with the dominant phyla in the roots of *Sinopodophyllum hexandrum* [32] and *Pennisetum sinese* [30], in the barks of *Eucommia ulmoides* [33], and the culturable fungal endophytes of *H*. *serrata* [18].

The dominant genera of endophytic fungi differed among the three tissues of the investigated *H*. *serrata* in our study. The genera of *Oidiodendron* and *Cladosporium* were dominant in the root and stem samples of the investigated *H*. *serrata*, respectively. Some strains of *Oidiodendron* were reported to promote nitrogen uptake and plant growth [34], and exhibit metal tolerance [35]. Some strains of *Cladosporium* exhibited an antimicrobial activity against *Bacillus cereus* IIIM 25 (Gram positive) and *Escherichia coli* ATCC 25922 (Gram negative) [36], and effectively reduced the infection of nematodes in some plant roots [37]. The genera of *Oidiodendron* and *Cladosporium* might confer similar benefit upon the host *H*. *serrata*. Some of endophytic fungi can produce antifungal active substances [38], which can improve their competitiveness and prevent colonization by other fungi. The tetranorlabdane diterpenoids from the extract of endophytic fungus *Botryosphaeria* sp. P483 isolated from *H*. *serrata* exhibited obviously antifungal activities against to *Gaeumannomyces graminis*, *Fusarium moniliforme*, *F*. *solani*, *F*. *oxysporum* and *Pyricularia oryzae* [11]. Interestingly, the genus of *Botryosphaeria* mainly existed in the root samples in our study, which might partly explain why the richness and diversity of endophytic fungi in the root samples were lower than that in the leaf and stem samples.

Endophytic fungi are considered as the fungi that live inside healthy plant tissues at a certain or whole stage of life cycle and do not cause obvious plant diseases [39]. Some endophytic fungi in the investigated *H*. *serrata* were identified as plant pathogenic fungi, such as the dominant genus *Phyllosticta* (11.56%) in the leaf samples. *Phyllosticta* was an important group of plant pathogenic fungi distributed worldwide that causes serious diseases, e.g., citrus and grapevine black spots [40, 41]. An abundant *Phyllosticta* in the leaf samples might be attributed to the investigated *H*. *serrata* leaves invaded by this pathogenic fungus, but that did not cause obvious lesions of the host plant temporarily. Another possible explanation was the limitation of the surface sterilization method used in this study, which was responsible for these identified plant pathogenic fungi as endophytes, because this surface sterilization method could not entirely remove the microorganisms adhered to the tissue surfaces.

Currently, about 12 fungal genera have been reported to produce huperzine A [15–17, 42–47], of which 7 genera were found in *H*. *serrata* in this study. Among them, *Cladosporium*, *Trichoderma*, and *Fusarium* with the relative abundance > 0.1% had the highest distribution in the stems, followed by the leaves and the roots, indicating that the endophytic fungi in the stems and the leaves were more involved in the synthesis of some secondary metabolites than those in the roots. Five of the reported fungal genera, that can produce huperzine A, were not detected in our study, which might be attributed to the investigated *H*. *serrata*, because the planting environment and age of host plant were important factors affecting the composition of its endophytic fungi. Therefore, it is necessary to further investigate the endophytic microorganisms of *H*. *serrata* from different growing environments and years, which were beneficial to obtain a comprehensive understanding of the endophyte community in *H*. *serrata*. Besides these endophytic fungi of the reported genera with an ability to produce huperzine, our results uncovered that *H*. *serrata* contained diverse endophytic fungi of yet unexplored potential importance, which a reservoir for developing endophyte resources for artificial cultivation *H*. *serrata* and production of useful bioactive compounds.

## Conclusion

In conclusion, this study reveals the community composition and structure of the endophytic fungi in the roots, stems, and leaves of *H*. *serrata*, and found endophytic fungal communities varying across the three tissues, and uncovered some dominant endophytic fungi in the three tissues, which benefit further scientific understanding of fungal community ecology in this medicinally important plant and better to tap functionally important endophytes.

## Supporting information

S1 Fig The length distribution of the effective tags in the root (R), stem (S) and leaf (L) samples. (TIF)

S2 Fig Heatmap displaying the relative abundances of the most dominant genera (relative abundance > 0.2%) in the root (R), stem (S) and leaf (L) samples. The dendrogram represents complete-linkage agglomerative clustering based on Euclidean dissimilarities.
(TIF)

S3 Fig Relative abundances of endophytic fungi at the genus level in the root (R), stem (S) and leaf (L) samples of *H*. *serrata*. (TIF)

S4 Fig Cluster pedigree diagrams showing the evolutionary relationships among the root (R), stem (S) and leaf (L) samples based on Binary-Jaccard. (TIF)

S1 Table The statistics and quality evaluation of the sequencing data. (DOCX)

S2 Table Distribution of fungal microbiome at class level in root, stem and leaf samples of *H*. *serrata*. (DOCX)

S3 Table Distribution of exclusive genera in the root, stem and leaf samples of *H*. *serrata*. (DOCX)
