# Supplementary figures and images for: Illumina-based analysis yields new insights into the diversity and composition of endophytic fungi in cultivated Huperzia serrata
Source: PLoS One. 2020 Nov 19;15(11):e0242258. doi: 10.1371/journal.pone.0242258 (PMC7676737; doi:10.1371/journal.pone.0242258)

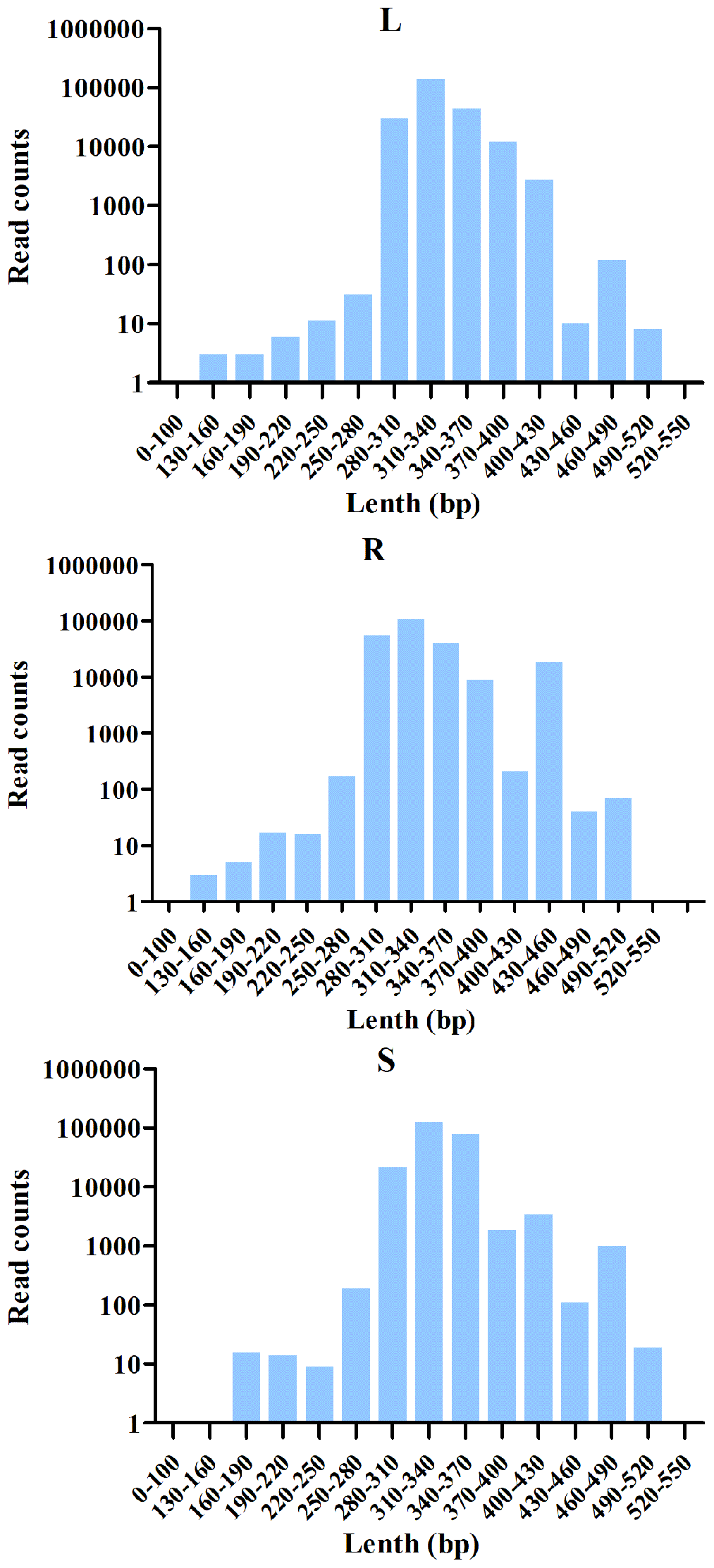

Supplement: S1 Fig — (TIF) [file pone.0242258.s001.tif]

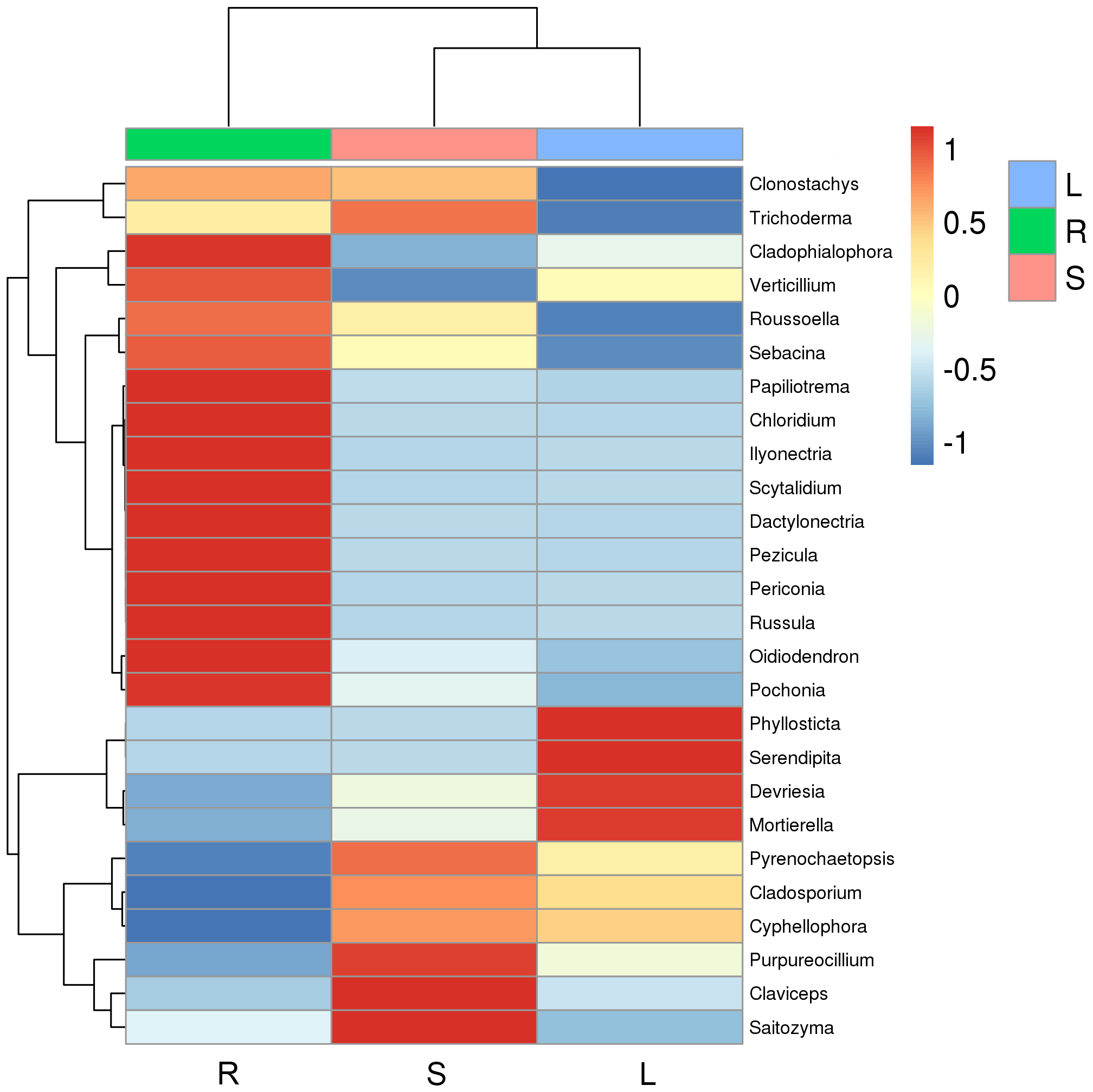

Supplement: S2 Fig — The dendrogram represents complete-linkage agglomerative clustering based on Euclidean dissimilarities. (TIF) [file pone.0242258.s002.tif]

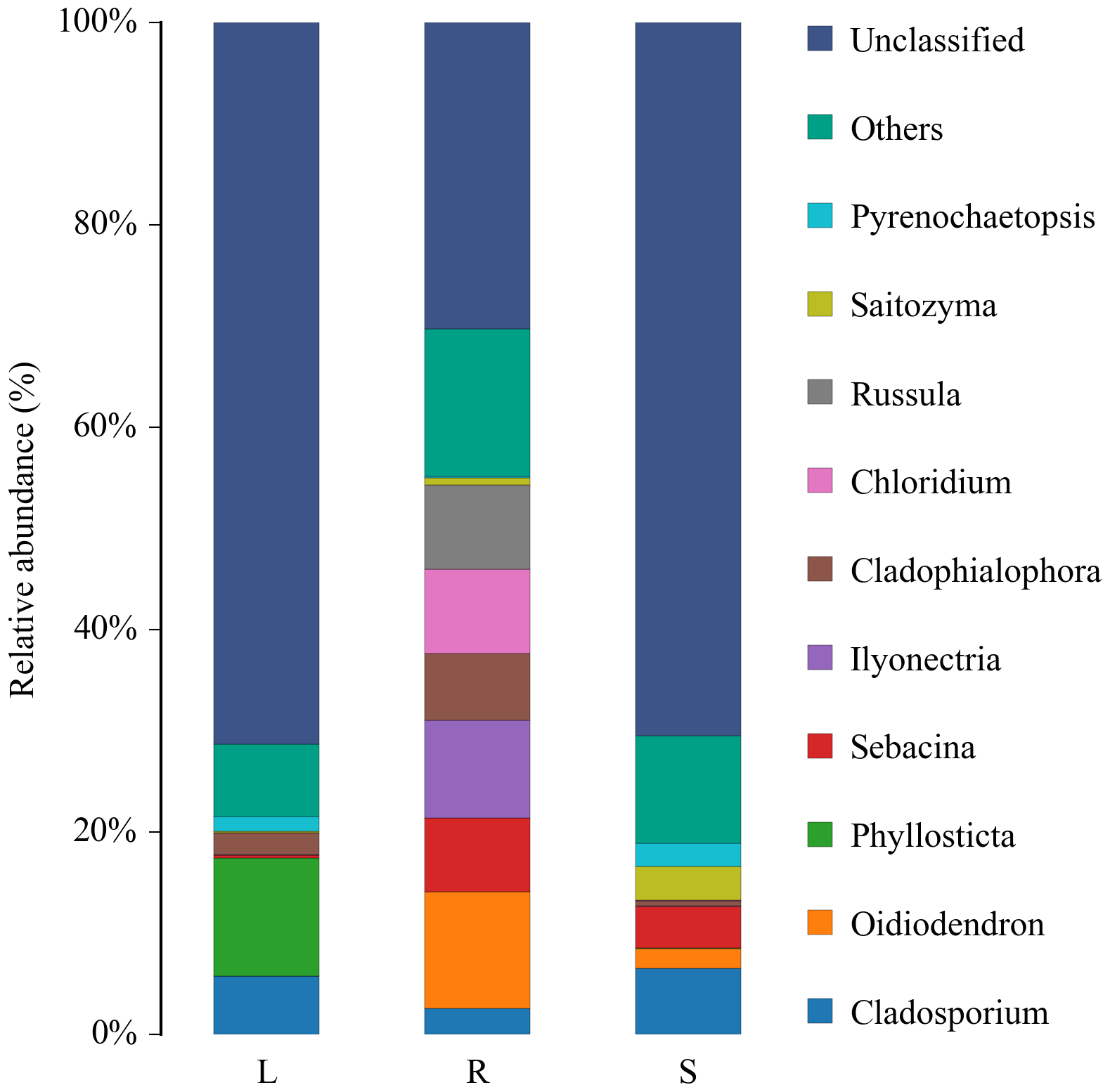

Supplement: S3 Fig — (TIF) [file pone.0242258.s003.tif]

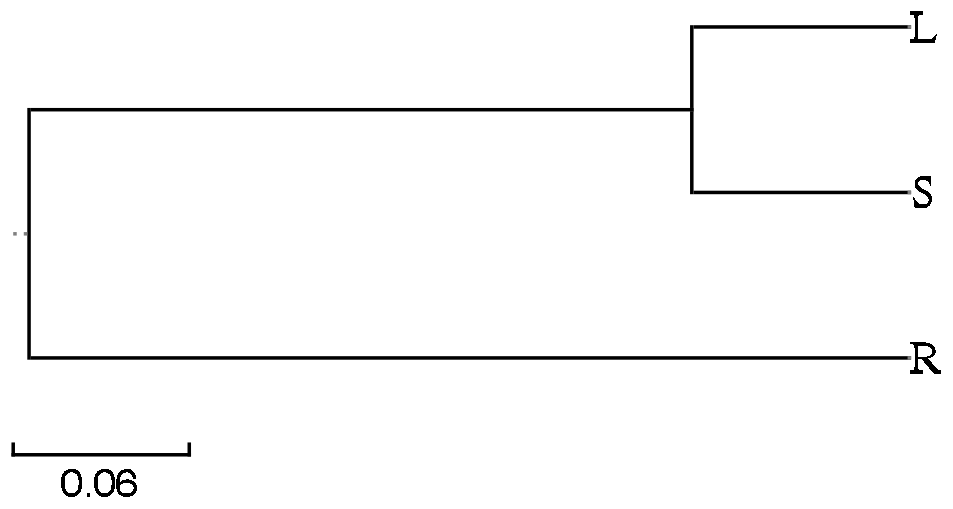

Supplement: S4 Fig — (TIF) [file pone.0242258.s004.tif]
