# Supplementary material for: Illumina-based analysis yields new insights into the diversity and composition of endophytic fungi in cultivated Huperzia serrata
Source: PLoS One. 2020 Nov 19;15(11):e0242258. doi: 10.1371/journal.pone.0242258 (PMC7676737; doi:10.1371/journal.pone.0242258)
Supplement: S1 Table — (DOCX) [file pone.0242258.s005.docx]

**S1 Table. The statistics and quality evaluation of the sequencing data.**

| **Sample origin** | **PE Reads** | **Raw Tags** | **Clean Tags** | **Effective Tags** | **AvgLen**  **(bp)** | **GC**  **(%)** | **Q20**  **(%)** | **Q30**  **(%)** | **Effective ratio**  **(%)** |
| --- | --- | --- | --- | --- | --- | --- | --- | --- | --- |
| Root | 239621 | 232390 | 232380 | 230078 | 304 | 54.31 | 99.51 | 98.81 | 96.02 |
| Stem | 239866 | 231195 | 231184 | 230283 | 303 | 52.58 | 99.55 | 98.88 | 96 |
| Leaf | 239778 | 232179 | 232170 | 231811 | 299 | 52.34 | 99.6 | 99.01 | 96.68 |
