# Supplementary material for: Illumina-based analysis yields new insights into the diversity and composition of endophytic fungi in cultivated Huperzia serrata
Source: PLoS One. 2020 Nov 19;15(11):e0242258. doi: 10.1371/journal.pone.0242258 (PMC7676737; doi:10.1371/journal.pone.0242258)
Supplement: S2 Table — (DOCX) [file pone.0242258.s006.docx]

**S2 Table. Distribution of fungal microbiome at class level in the roots, stems, and leaves.**

| **Phylum** | **Classes** | **Relative abundance (%)** | | |
| --- | --- | --- | --- | --- |
|  |  | **Roots** | **Stems** | **Leaves** |
| Ascomycota | Dothideomycetes | 14.71 | 21.92 | 37.02 |
|  | Eurotiomycetes | 10.01 | 8.17 | 4.05 |
|  | Geoglossomycetes | 0.002 | 0.02 | 0.0005 |
|  | Lecanoromycetes | 0.0004 | 0.02 | 0.05 |
|  | Leotiomycetes | 17.03 | 2.07 | 0.12 |
|  | Orbiliomycetes | 0.01 | 14.77 | 2.26 |
|  | Pezizomycetes | 0 | 0.02 | 0.002 |
|  | Sordariomycetes | 26.47 | 14.88 | 3.79 |
| Basidiomycota | Agaricomycetes | 15.85 | 4.64 | 9.81 |
|  | Agaricostilbomycetes | 0 | 0.16 | 0.02 |
|  | Cystobasidiomycetes | 0 | 0.21 | 0.14 |
|  | Exobasidiomycetes | 0 | 0.02 | 0.003 |
|  | Microbotryomycetes | 0.0004 | 0.05 | 0.02 |
|  | Tremellomycetes | 1.29 | 4.72 | 0.99 |
|  | Ustilaginomycetes | 0 | 0.07 | 0.12 |
| Chytridiomycota | Spizellomycetes | 0 | 0.01 | 0.004 |
| Mortierellomycota | Mortierellomycetes | 0.01 | 0.18 | 0.60 |
| Mucoromycota | Umbelopsidomycetes | 0.0004 | 0.03 | 0.02 |
| Glomeromycota | Glomeromycetes | 0.07 | 0 | 0.0005 |
| Olpidiomycota | GS17 | 0 | 0.12 | 0.02 |
|  | GS18 | 0.001 | 0.05 | 0 |
