# Supplementary material for: Illumina-based analysis yields new insights into the diversity and composition of endophytic fungi in cultivated Huperzia serrata
Source: PLoS One. 2020 Nov 19;15(11):e0242258. doi: 10.1371/journal.pone.0242258 (PMC7676737; doi:10.1371/journal.pone.0242258)
Supplement: S3 Table — (DOCX) [file pone.0242258.s007.docx]

**S3 Table.** **Distribution of exclusive genera in the root, stem and leaf samples of *H. serrata*.**

| **Phyla** | **Class** | **Genus** | **Relative abundance (%)** | | |
| --- | --- | --- | --- | --- | --- |
|  |  |  | **Roots** | **Stems** | **Leaves** |
| Ascomycota | Dothideomycetes | *Botryosphaeria* | 0.09 | 0 | 0 |
|  | Eurotiomycetes | *Phialophora* | 0 | 0.02 | 0 |
|  |  | *Scytalidium* | 0.03 | 0 | 0 |
|  | Orbiliomycetes | *Lecophagus* | 0 | 0.02 | 0 |
|  | Sordariomycetes | *Idriella* | 0.13 | 0 | 0 |
| Basidiomycota | Agaricomycetes | *Clavaria* | 0 | 0.08 | 0 |
|  |  | *Peniophora* | 0 | 0.02 | 0 |
